# Plug and play virus-like particles for the generation of anti-toxin antibodies

**DOI:** 10.1016/j.toxcx.2024.100204

**Published:** 2024-08-26

**Authors:** Rebecca J. Edge, Amy E. Marriott, Emma L. Stars, Rohit N. Patel, Mark C. Wilkinson, Lloyd D.W. King, Julien Slagboom, Choo Hock Tan, Kavi Ratanabanangkoon, Simon J. Draper, Stuart Ainsworth

**Affiliations:** a Department of Infection Biology and Microbiomes, Institute of Infection, Veterinary and Ecological Sciences, University of Liverpool, Liverpool, L3 5RF, United Kingdom; b Centre for Snakebite Research and Interventions, Department of Tropical Disease Biology, Liverpool School of Tropical Medicine, Pembroke Place, Liverpool, L3 5QA, United Kingdom; c Department of Biochemistry, University of Oxford, Dorothy Crowfoot Hodgkin Building, Oxford, OX1 3QU, United Kingdom; d Kavli Institute for Nanoscience Discovery, University of Oxford, Dorothy Crowfoot Hodgkin Building, Oxford, OX1 3QU, United Kingdom; e Amsterdam Institute of Molecular and Life Sciences, Division of BioAnalytical Chemistry, Department of Chemistry and Pharmaceutical Sciences, Faculty of Science, Vrije Universiteit Amsterdam, De Boelelaan 1085, Amsterdam, 1081HV, the Netherlands; f School of Medicine, College of Life Sciences and Medicine, National Tsing Hua University, Hsinchu, 300, Taiwan; g Institute of Bioinformatics and Structural Biology, National Tsing Hua University, Hsinchu, 300, Taiwan; h Department of Pharmacology, Faculty of Medicine, University of Malaya, Kuala Lumpur, 50603, Malaysia; i Department of Microbiology, Faculty of Science, Mahidol University, Bangkok, 10400, Thailand

## Abstract

Snakebite is a major global health concern, for which antivenom remains the only approved treatment to neutralise the harmful effects of the toxins. However, some medically important toxins are poorly immunogenic, resulting in reduced efficacy of the final product. Boosting the immunogenicity of these toxins in the commercial antivenom immunising mixtures could be an effective strategy to improve the final dose efficacy, and displaying snake antigens on Virus-like particles (VLPs) is one method for this. However, despite some applications in the field of snakebite, VLPs have yet to be explored in methods that could be practical at an antivenom manufacturing scale. Here we describe the utilisation of a “plug and play” VLP system to display immunogenic linear peptide epitopes from three finger toxins (3FTxs) and generate anti-toxin antibodies. Rabbits were immunised with VLPs displaying individual consensus linear epitopes and their antibody responses were characterised by immunoassay. Of the three experimental consensus sequences, two produced antibodies capable of recognising the consensus peptides, whilst only one of these could also recognise native whole toxins. Further characterisation of antibodies raised against this peptide demonstrated a sub-class specific response, and that these were able to elicit partially neutralising antibody responses, resulting in increased survival times in a murine snakebite envenoming model.

## Highlights

• Three finger toxins (3FTx) in snake venom are poorly immunogenic.
• Virus-like particles (VLP) could be used to boost immunogenicity.
• Rabbits were immunised with plug and play VLPs decorated with 3FTx linear peptides.
• VLP-peptide antibodies could recognise sub-class specific 3FTxs from multi genera.
• Antibodies against VLP-peptides were partially neutralising in an *in vivo* mouse model.

## Graphical abstract

**Figure:**
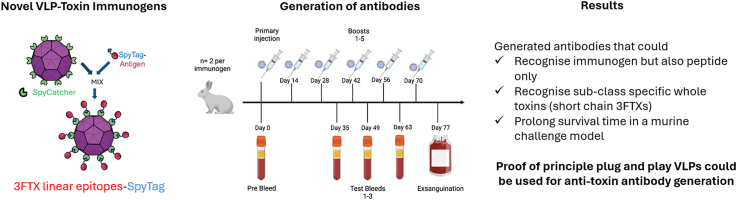

## 1 Introduction

Snakebite envenoming (SBE) is a major global cause of mortality and morbidity, affecting upwards of two million people per year and resulting in a recently estimated death toll exceeding 63,000 people in 2019 alone (GBD 2019, 2022). Venoms are a complex mixture of proteins, although there are several distinctive medically important toxin families primarily driving pathology (Casewell et al., 2020; Gutiérrez et al., 2017). The only specific treatment for SBE is antivenom, a polyclonal antibody-based serotherapy raised by immunising large animals with venom in order to produce anti-toxin antibodies (WHO Expert Committee on Biological, 2017; León et al., 2018). However, there are numerous caveats to antivenom use such as poor inter- and intra-species recognition of toxins (Casewell et al., 2010), adverse reactions ranging from mild through to severe anaphylaxis (de Silva et al., 2016), and some medically important toxins, particularly low molecular weight three finger toxins (3FTxs) and phospholipase_2_ (PLA_2_) toxins, having poor immunogenicity resulting in reduced weaker efficacy compared to the neutralisation of larger toxins more commonly associated with coagulopathies (León et al., 2011; Pruksaphon et al., 2022; Chan et al., 2023). In elapid venoms, both 3FTxs and PLA_2_ are the most abundant toxin families, and 3FTxs have been identified across all species (Tasoulis and Isbister, 2023). Alpha-neurotoxins are a particularly important sub-class of 3FTx, competing for nicotinic acetylcholine receptors and preventing function. This competitive inhibition ultimately prevents normal neurotransmission, resulting in descending paralysis, respiratory failure and death if adequate and timely treatment is not provided (Nys et al., 2022). Given the aforementioned efficacy issues against these toxins, there is a requirement to administer large doses of antivenom, further confounding the risk of adverse reactions and socioeconomic cost implications.

New strategies for the treatment of SBE are currently under investigation, such as monoclonal or recombinant antibodies and alternative antibody formats, such as camelid V_H_H, to replace traditional antivenom (Fernandes et al., 2010; Julve Parreño et al., 2018; Laustsen et al., 2017; Prado et al., 2016; Richard et al., 2013), and small molecule therapeutics to complement or replace antibody-based therapies (Albulescu et al., 2020; Chowdhury et al., 2021; Villalta-Romero et al., 2017; Xie et al., 2020). Whilst these new alternatives to traditional antivenom are highly promising, they currently remain at an early stage of translational development. An alternative strategy, which in theory could be rapidly implemented and may serve as an interim or long term improvement, is to move away from using crude venom as an immunogen in antivenom manufacture and instead focus on the development of rationally tailored immunogens. Given that these proposed improvements will not alter the physical characteristics of the final antivenom product, which will remain a polyclonal antivenom mixture, such improvements may be rapidly implemented as such improved antivenom products would likely avail of the unique regulatory frameworks in which antivenoms are currently assessed.

One such approach is to use Virus-like particles (VLPs) to improve the antigenicity and manufacturing animal immune responses towards key venom toxins. VLPs are non-infectious nanostructures with an established history and diverse range of therapeutic applications, from vaccine scaffolds to pharmaceutical delivery platforms (Mohsen and Bachmann, 2022). We have previously used recombinant hepatitis B core antigen (HBcAg) VLPs displaying a genetic fusion of linear venom-toxin epitopes to rapidly generate antibodies in mice capable of recognising regionally distinct elapid venoms (Menzies et al., 2022). However, this approach, necessitating genetic fusion and therefore recombinant generation of multiple VLP-epitopes requiring extensive optimisation and purification, proved highly challenging and would ultimately be unfeasible in industrial application. The development of SpyCatcher-SpyTag ‘plug and play’ VLP systems presents an opportunity to overcome the need for distinct VLP-toxin expressions, instead requiring a universal VLP carrier which can be simply decorated with antigens harbouring a corresponding tag (Bruun et al., 2018). This format enables flexibility in the use of chemically synthesised linear peptide epitopes, substantially streamlining production and assessment of potential antigens.

The use of linear peptides as antigens in antivenom manufacture provides several advantages given their lack of toxicity, their ease of production and their stability (Felicori et al., 2009). Linear epitopes in particular are more readily identifiable and cheaper to produce than conformational antigens, increasing the likelihood this method of generating anti-toxin antibodies could be translational to manufacturing practice. There is also now a significant amount of research demonstrating their utility for anti-toxin antibody generation, and subsequent neutralisation of venom specific pathologies (Menzies et al., 2022; Bermúdez-Méndez et al., 2018; Ramos et al., 2016; Castro et al., 2015; Molina et al., 2018; Mendes et al., 2013).

Here, we describe the development of highly conserved and immunogenic linear peptide epitopes empirically deduced from short-chain α-neurotoxin 3FTxs (sc3FTx) using high density peptide arrays, and their subsequent use coupled to SpyCatcher VLPs to generate anti-toxin antibodies in rabbits. The specificity and neutralising capabilities of the subsequent polyclonal antibodies were evaluated, resulting in increased survival times in a murine SBE model.

## 2 Methods

### 2.1 Toxin sequences

Searches on Genbank and Uniprot using the terms “three finger toxin”, “3FTX” and “alpha-neurotoxin” were performed. Amino acid sequences of search results were downloaded and compiled into a multi-FASTA file. Sequences were then scrutinised using BLAST to remove any 3FTx sequences that did not represent sc3FTx. This process resulted in a dataset consisting of 323 individual sc3FTx sequences representing 40 species. Duplicate sequences where sc3FTx are conserved 100% at the amino acid level across species were removed, resulting in a final data set of 152 unique sequences (Supplementary File S1).

### 2.2 Peptide arrays

Both the manufacture of sc3FTx peptide arrays and subsequent linear epitope mapping was performed by PEPperPRINT, Germany. The sc3FTx sequences were split into linear 15 amino acid peptides with a peptide-peptide overlap of 14 amino acids. Resulting peptides with redundancy within the dataset were removed. The resulting peptide microarrays consisted of 17,401 different peptides printed in duplicate and were framed by additional HA (YPYDVPDYAG, 739 spots) control peptides.

### 2.3 Antivenoms

The following equine antibodies and antivenoms were used to probe the peptide microarrays. Commercial antivenoms were chosen based on their established ability to neutralise elapid venom induced neurotoxic pathology in preclinical envenoming (Vargas et al., 2011; Soopairin et al., 2023; Gopal et al., 2024; Ainsworth et al., 2020). Naïve Equine IgG (BioRad), SAIMR polyvalent (South African Vaccine Producers, batch BF00546, Expiry: 11-January-2017, FAV Afrique (Sanofi, Batch K8453, Expiry: 01-June-2013), PANAF (Premium Serums, Batch PANAF-008, Expiry: 01-December-2023), Premium India (Premium Serums, Batch: ASVS-1/LY024), TRC Neuro Polyvalent (Thai Red Cross, NP00120, Expiry: 27-March-2020), ICP Anticoral (ICP, Batch: 5480914ACLQ, Expiry: 09-January-2017), CSL polyvalent (Sequris, Batch: BO55318601, Expiry: 01-October-2016) and an equine experimental anti-neurotoxic polyclonal sera previously described (Ratanabanangkoon et al., 2020). Further details on antivenoms (format, concentration, indication) can be found in Supplementary File S2, Table S1.

### 2.4 Toxins

The purified native toxins used in this study were sourced commercially or purified in-house from whole venom obtained from specimens maintained in the herpetarium at the Liverpool School of Tropical Medicine (Table 1). The full methods for the in-house purifications and subsequent mass spectrometry identification can be found in Supplementary File S2.

**Table 1:** Purified three finger toxins (3FTx) and control phospholipase A2 (PLA_2_) used in this study. The species whose venom each toxin was purified from, their subtype, the source of the toxin and their protein accession numbers are detailed.

Toxin	Species	Subtype	Source (Cat #)	Protein accession number
α-bungarotoxin	*Bungarus multicintus*	Long	Invitrogen (B1601)	3L21A_BUNMU
α-elapitoxin Dpp2d	*Dendroaspis polylepis*	Long	LSTM	3L24_DENPO
Alpha-cobratoxin, IIα	*Naja kaouthia*	Long	LSTM	3L21_NAJKA
short neurotoxin 1	*Naja pallida*	Short	LSTM	3S11_NAJPA
short neurotoxin 1	*Dendroaspis jamesoni kaimosae*	Short	LSTM	3S11_DENJA
short neurotoxin 1	*Naja philippinensis*	Short	LSTM	3S11_NAJPH
Erabutoxin a	*Laticauda semifasciata*	Short	Latoxan (L8110)	3S1EA_LATSE
Erabutoxin b	*Laticauda semifasciata*	Short	Latoxan (L8111)	3S1EB_LATSE
Cytotoxin-1 3FTx	*Naja pallida*	P-type	LSTM	3SA1_NAJPA
PLA_2_	*Naja nigricollis*	Acid and Basic 1:1	LSTM	PA2A1_NAJMO and PA2B4_NAJNG

LSTM: Liverpool School of Tropical Medicine.

### 2.5 Epitope discovery and consensus peptide generation

Consensus peptides were designed by interrogating peptide array results, specifically examining all peptides which provided a substantial signal (above a threshold) vs. each antivenom. Thresholds were arbitrary (higher/lower) for different antivenoms based on their overall performance in recognising arrays. Epitopes which were recognised by antivenoms but also by naïve equine IgG were removed. The remaining peptides were clustered into epitope regions based on sequence similarity using CD-HIT (Fu et al., 2012) at 40% similarity. Epitope regions were mapped onto a solved sc3FTx structure from *Dendroapsis polylepis* (PDB identifier: 1NTX) (Labhardt et al., 1988) using ChimeraX (Pettersen et al., 2021). Alignments and amino acid conservation analysis were performed in MEGA 11 (Tamura et al., 2021). All sequences within cluster were then used to generate a consensus epitope sequence using WebLogo (Crooks et al., 2004) using default settings. For positions within consensus sequences which displayed heterologous conserved amino acids, the most common variant was used.

### 2.6 Peptide synthesis

Peptides were synthesised by Peptide synthetics (UK). Each peptide consisted of the N-terminal respective consensus sequence, followed by a glycine serine linker (GSGGSGGSG), followed by a C-terminal SpyTag (GAHIVMVDAYKPTK). Purity of each peptide was >75%. Peptides were stored at −20 °C until required.

### 2.7 Coupling of VLP to peptides

The mi3-SpyCatcher (mi3-SC) VLP used in this study was produced at the University of Oxford as previously described by Bruun et al. (2018), and was expressed in an *Escherichia coli* expression system and purified from cell lysate by C-tag affinity selection and size exclusion chromatography. Consensus peptides were reconstituted in water and coupled individually to mi3-SC VLP at a 3x molar excess of peptide as per Bruun et al. (2018). Briefly, 30 μM of peptide was coupled to 10 μM of mi3-SC in 25 mM Tris-HCl pH 8.5150 mM NaCl overnight at 25 °C in 150 μL reactions. Reaction mixtures were pooled by peptide and dialysed in PBS-0.1% Tween 20 in 300 kDa MWCO dialysis devices for 24 h with two buffer changes. Coupling was confirmed by size increase of mi3-SC by reducing SDS-PAGE with Coomassie staining (4–20% gel, 200V, 25 min, BioRad TGX system). Coupled peptide-mi3-SC was then stored at −80 °C in individual dose aliquots until immunisation.

### 2.8 Generation of polyclonal sera

Polyclonal antibody production was performed by Antibody Production Services (a division of Life Science Group Ltd., UK) in accordance with the UK Animals (Scientific Procedures) Act 1986. *Oryctolagus cuniculus* (European rabbits) were housed in Tecniplast 4P02B700 cages using filtered air, with food and water ad libitum, natural light cycles and regulated temperature. Rabbits were acclimatised for at least 14 days prior to use, and were aged between 16 and 20 weeks (3 kg minimum weight) at the beginning of the study. The rabbits were immunised with either mi3-SC alone, or mi3-SC coupled to C6, C10 or C11 peptide with and without the presence of adjuvant (n = 2 per immunogen and adjuvant condition, n = 16 in total). A standard 77-day immunisation protocol was carried out consisting of a primary injection of 50 μg of immunogen with or without Freund's complete adjuvant (FCA), followed by 5 × 50 μg boosts with or without Freund's incomplete adjuvant (FIA) at two-week intervals. Test bleed samples of 10 mL were collected from the rabbits prior to immunisation as well as on days 35, 49 and 63, which were all performed whilst rabbits were sedated via the mid line artery of ear. Rabbits were exsanguinated and terminal blood collected on day 77. Blood was kept at room temperature overnight to allowing clotting, then centrifuged at 5020g for 5 min under aseptic conditions and placed in sterile vials for same day shipping on wet ice. Sera was then stored at −20 °C until IgG purification was performed using rProtein A GraviTrap columns, eluting in 3x1 mL fractions to generate a concentrated middle fraction of between 30 and 40 mg/mL for each sample The subsequent purified IgG was then stored at −20 °C until required for downstream analysis.

### 2.9 ELISA

To assess the seroconversion of the rabbits, ELISA experiments were performed with the purified rabbit IgG against the immunogen, peptide only and whole toxins. Nunc MaxiSorp ELISA plates were coated with 100 ng per well of the relevant protein or peptide in 50 mM carbonate-bicarbonate coating buffer (pH 9.5) and incubated at 37 °C for 1 h. The plates were then washed 6 times with TBS-0.1% Tween 20 (TBS-T) before being blocked with 5% milk in TBS-T for 3 h. The plates were washed three times before rabbit IgG or relevant controls (naïve rabbit IgG, no primary/no secondary antibody wells) were added and incubated overnight at 2–8 °C. For the time course ELISAs of sample bleeds and terminal bleeds against peptide only, starting dilutions of 1:100 in 5% milk in TBS-T were then 5-fold serial diluted down to 1:2500 and 1:12500. For the terminal bleeds against immunogen, the dilution series remained 5-fold but began at 1:500 with six further dilutions down to 1:1.56 × 10^6^. Finally, the terminal bleeds against whole toxin used a dilution series starting at 1:50, five-fold diluted to 1:250 and 1:1250. All naïve control IgG samples, as well as mi3-SC only antibodies when used to confirm peptide specific responses, were used at the starting sample dilution on the plate. Following overnight incubation, the plates were washed six times before anti-rabbit IgG secondary antibody conjugated to horseradish peroxidase was added at a 1:6000 dilution in PBS. Secondary antibody incubation was carried out for 2 h at room temperature before plates were washed six times again. The ELISAs were then developed with 0.1 mg/mL 2,2′-azino-bis[3-ethylbenzthiazoline-6-sulfonic acid] diammonium salt substrate in 0.05 M citrate buffer pH 5.0 with 0.0075% (v/v) hydrogen peroxide for 25 min before the optical density (OD) was read at 405 nm. All samples were measured in duplicate, and every plate included relevant antibody controls. Results were interrogated for statistical significance by ordinary one-way ANOVA and multiple comparisons tests using Graphpad Prism 10.2.3, using the starting dilution data except in the instance of evaluating the peptide verses mi3-SC specific response when the 1:12500 dilution was used.

### 2.10 Immunoblots

Immunoblot analysis of generated rabbit polyclonal sera were carried out on a small panel of whole toxins. SDS-PAGE electrophoresis was performed by incubating short neurotoxin 1 (*N. pallida*), Erabutoxin a (*L. semifasciata*) and a phospholipase A2 (*N. nigricollis*) at 85 °C for 5 min with equal volume 2x denaturing protein loading buffer with 10% DTT, then 1.8 μg of sample was run at 200V on a 4–20% Mini-Protean TGX gel (BioRad). The proteins on the gel were transferred onto a nitrocellulose membrane using a TransBlot TURBO mini system and stained with Revert 700 Total Protein Stain as per manufacturer protocol. The proteins were imaged using the 700 nm channel on an Odyssey FC imaging system and then destained (0.1 M sodium hydroxide, 30% (v/v) methanol in H_2_O) before proceeding with the immunoblotting protocol. The membranes were blocked with 5% fat-free milk powder in TBS-T for 3 h, the probed with a 1:1000 dilution of the relevant experimental rabbit IgG overnight at 2–8 °C on a rocking platform. The membranes were washed four times for 5 min with TBS-T and then incubated with donkey anti-rabbit IRDye 800 at a 1:15,000 dilution for 2 h at room temperature. Finally, the membranes were washed three times for 5 min with TBS-T and then once with TBS only, before imaging using the 700 nm and 800 nm channel on an Odyssey FC imaging system. IgG samples from naïve rabbits or those immunised by mi3-SC alone were ran as controls.

### 2.11 Neutralisation of neurotoxin induced lethality

#### 2.11.1 Ethical approvals

Murine animal experiments were conducted under protocols approved by the Animal Welfare and Ethical Review Boards of the Liverpool School of Tropical Medicine and the University of Liverpool, as per project license PP2669304 approved by the UK Home Office in accordance with the UK Animal (Scientific Procedures) Act 1986.

#### 2.11.2 Animal maintenance

CD1 mice (male, 18–20 g, Charles River UK) were grouped in cages of five upon arrival and acclimated for one week before experimentation in specific pathogen-free conditions. Holding room conditions were 23 °C with 45–65% humidity and 12/12-h light cycles (350 lux). Mice were housed in Tecniplast GM500 cages (floor area 501 cm^2^) containing 120 g Lignocell wood fibre bedding (JRS, Germany), Z-nest biodegradable paper-based material for nesting, and environmental enrichment (red house, clear polycarbonate tunnel and loft). Mice had ad libitum access to irradiated PicoLab food (Lab Diet, USA) and reverse osmosis water in an automatic water system.

#### 2.11.3 *In vivo* procedure

Mice (29.7g mean weight, range 26–33g) were provided analgesia (morphine, 1.25 mg/kg) via subcutaneous injection 15 min prior to toxin challenge. Five groups of five mice were used (in total 25 mice). All groups received a toxin challenge consisting of 4 μg (approximately 2 x LD_50_ (Karlsson et al., 1966) of short neurotoxin 1 purified from the venom *N. pallida* in PBS which had been pre-mixed and incubated for 30 min at 37 °C with the following: group 1; PBS only (no intervention control), group 2; 2 mg of anti-peptide antibodies, group 3; 4 mg of anti-peptide antibodies, group 4; 8 mg of anti-peptide antibodies, group 5; 8 mg of SAIMR polyvalent (BF00546, expiry January 11, 2017). Total injection volume was 200 μl which was administered via the tail vein. Mice were continuously monitored for 60 min and were euthanised via cervical dislocation upon progression to humane endpoint (loss of self-righting reflex). Any surviving mice at the end of the experiment were euthanised by cervical dislocation. All experiments used mixed gender experimenters and experimenters were unblinded to the test articles. Kaplan-Meir survival curves were statistically compared using the log-rank test for trend in Graphpad Prism 10.0.3.

## 3 Results

### 3.1 Linear toxin epitope discovery and antigen design

**Fig. 1:**
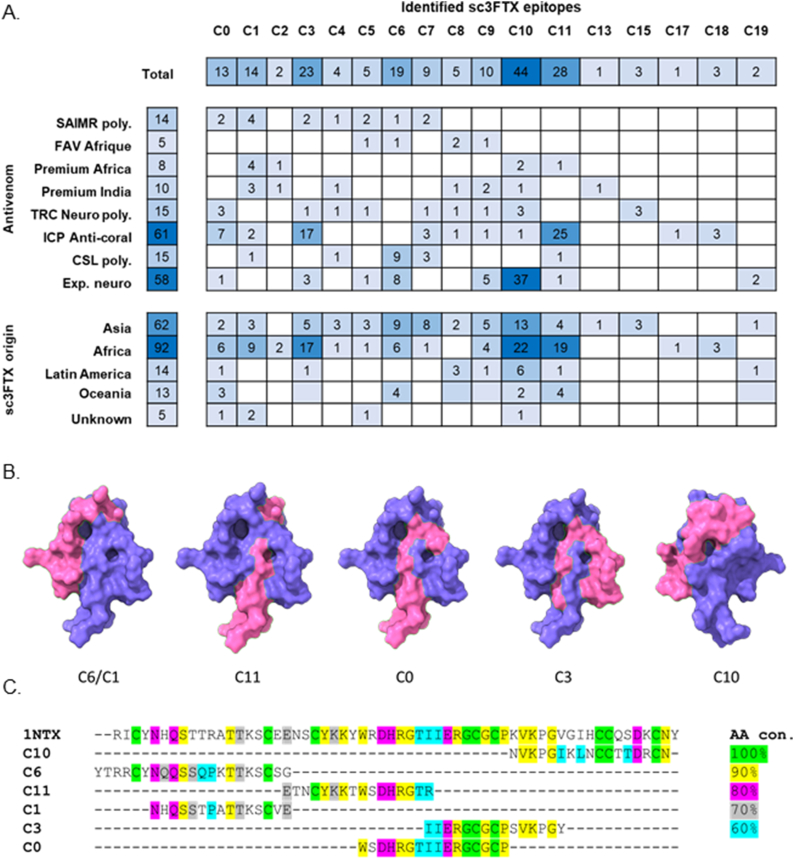
**Frequency, distribution and features of detected alpha short chain three finger toxin (sc3FTX) epitopes**. A) Heat maps demonstrating frequency (number displayed in each cell) in which each epitope (C0-C18) is recognised by antivenoms (top) or is recognised based on the geographic origin of the sc3FTx (fewer = light blue, greater = dark blue). Blank cells represent no recognition. B) Mapping and localization of selected epitopes onto a 3D model of a sc3FTx 1NTx from *D. polylepis* (PDB = 1NTx). C) Alignment and amino acid (AA) conservation of epitopes vs. 1NTx. Differences in AA conservation at individual residues amongst all sc3FTx sequences within an epitope region are represented by colour code (legend). Resides without colour demonstrates that particular residue is not representative or conserved within the data set. (For interpretation of the references to colour in this figure legend, the reader is referred to the Web version of this article.)

Probing of peptide arrays representing 152 unique sc3FTx amino acid sequences with eight antivenoms/sera indicated/developed for elapid envenoming revealed 17 distinct epitope regions (Fig. 1A, Supplementary File S3). Frequency of recognition of each epitope varied substantially, from a recognition by a single antivenom towards a specific sc3FTx, to recognition of an epitope by several antivenoms/sera across multiple sc3FTxs (Fig. 1a). Epitope recognition was not confined to the specific origin of the scNTx and antivenom/sera, although there was preferential recognition of an epitope based on geography in some instances. However, this needs to be viewed in the context of the bias towards African and Asian sc3FTxs within the assembled sc3FTx sequences, with recognition of epitopes in sc3FTx from the Americas and Oceania notably less frequent (Fig. 1A). Examples include C3 and C11 being recognised more frequently in sc3FTXs of African origin and C10 being recognised more frequently in sc3FTx of Asian origin. Notably, ICP Anti-coral, an antivenom manufactured for neutralising envenoming of *Micrurus* in north and central America, seemed particularly adept at recognising sc3FTXs of African origin (Fig. 1A).

**Fig. 2:**
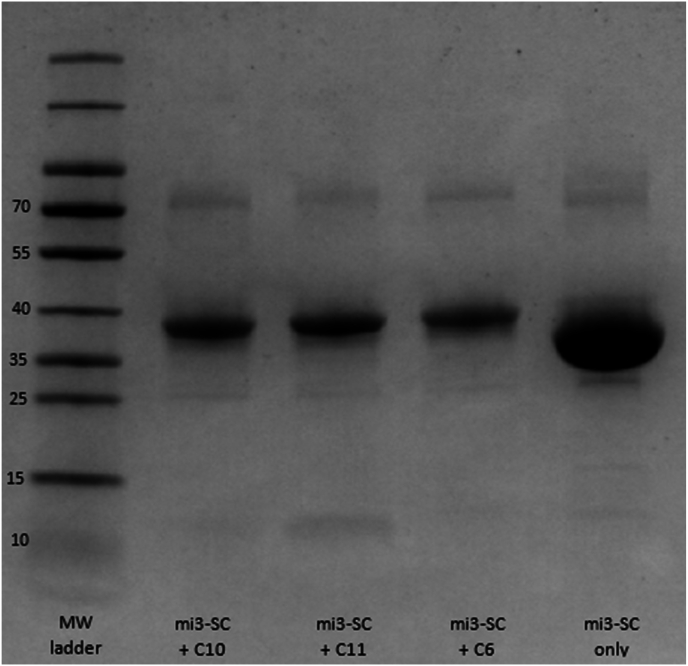
**Coupling efficiency of mi3-SC-peptide complexes for rabbit immunogens**. Peptide and mi3-SC were coupled together at a molar ratio of 1:3, then efficient coupling was demonstrated by an increase in size from mi3-SC alone using SDS-PAGE. These formed the final immunogens for rabbit immunisation schedule.

Six epitopes were recognised with notably higher frequency; C0, C1, C3, C6, C10 and C11. All epitopes had varying degrees of overlap with other epitopes, and when mapped to a typical sc3FTx appeared to be primarily found on the defining structural ‘finger’ features of sc3FTxs, including C1/C6 (first finger), C0/C11 (second finger) and C10 (third finger) (Fig. 1B). Epitope C3 covers part of finger 2, through the ‘core’ of the molecule and into finger 3. Based on frequency of recognition of epitopes, the distinct surface regions in which they resided on sc3FTxs, and their amino acid conservation (Fig. 1C), epitope regions C6, C10 and C11 were selected for further development for immunisation. Sequences corresponding to these regions were analysed to generate a consensus sequence for each epitope region, which was subsequently chemically synthesised (Supplementary File S2, Table S2.) along with a SpyTag for coupling to mi3-SC VLPs (Fig. 2).

### 3.2 Evaluation of anti-consensus toxin polyclonal sera

**Fig. 3:**
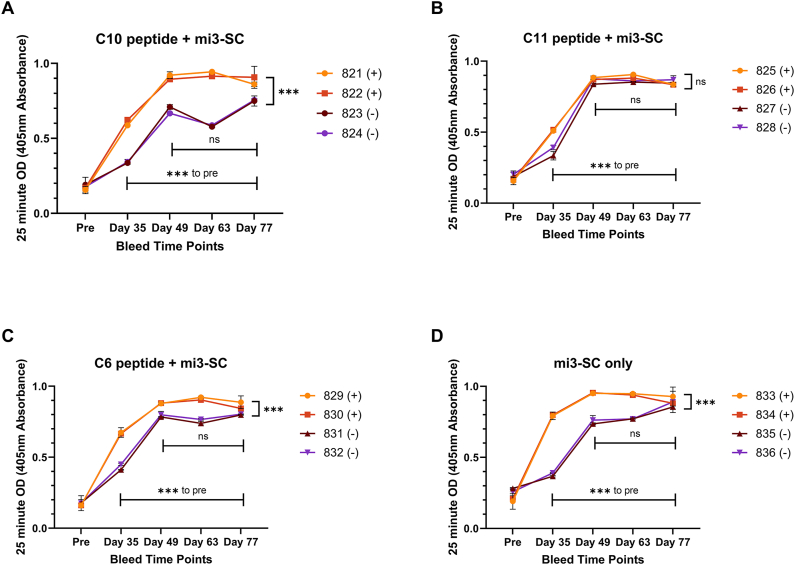
**ELISA time course of rabbit IgG response to VLP immunogens.** The ability of rabbit IgG to recognise the corresponding VLP immunogen over time was determined by ELISA for each individual rabbit (A; C10, B; C11, C; C6 and D; mi3-SC alone). The (+) denotes rabbits who received immunogen plus adjuvant, the (−) refers to the rabbits who received immunogen alone (n = 2 per experimental condition). Each sample was tested in duplicate at 1:2500 dilution, and data points show mean ± standard deviation of the individual rabbit results. Statistical results demonstrate analysis between pre sample and experimental time points, between experimental time points, and within adjuvant or no adjuvant groups.

**Fig. 4:**
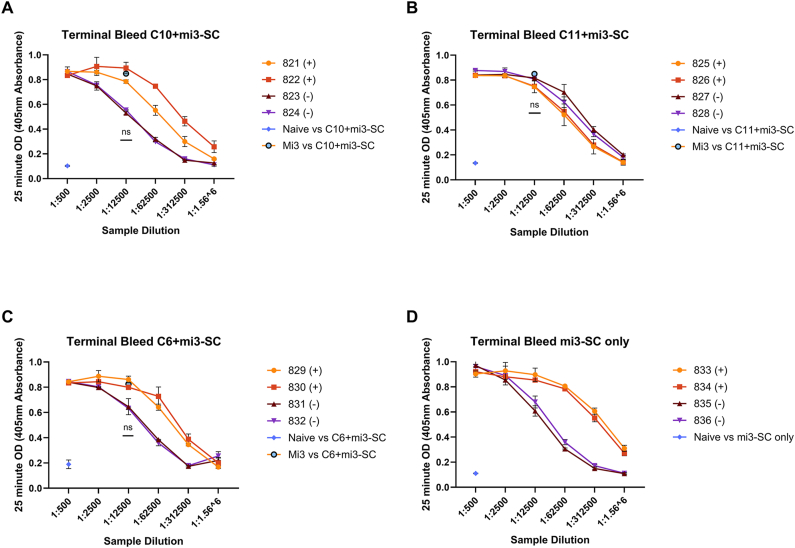
**The titre of rabbit IgG against VLP immunogens by endpoint titration ELISA.** A five-fold titration curve of IgG purified from terminal bleeds was performed against the corresponding VLP immunogen starting at 1:500 dilution (A; C10, B; C11, C; C6 and D; mi3-SC alone). The (+) denotes rabbits who received immunogen plus adjuvant, the (−) refers to the rabbits who received immunogen alone (n = 2 per experimental condition). A naïve rabbit IgG pool (n = 8) was tested against each immunogen at a 1:500 dilution, and pooled IgG from rabbits immunised with mi3-SC alone (n = 4) was also tested against each peptide-mi3-SC immunogen at a 1:500 dilution. Each sample was tested in duplicate, and data points show mean ± standard deviation of the individual rabbit results. Statistical results demonstrate analysis between individual rabbit antibody titres at 1:12500 dilution against their own immunogen and against mi3-SC only.

Antibody recognition of the immunogen was observed by ELISA in all rabbits within each experimental group, peaking at day 49 and remaining high for the remainder of the time course (Fig. 3A–D, p ≤ 0.0003 for all timepoints to baseline, p > 0.05 between days 49–77, for all immunogens). On average, rabbits that also received the adjuvant had slightly higher titres at each timepoint than rabbits that received immunogen only (Fig. 3A, C, 3D, p ≤ 0.0004 for all paired timepoints across C10, C6 and mi3-SC immunogens, except for mi3-SC day 77 p > 0.05). However, this wasn't constant across all experimental groups, with the C11 immunogen looking comparable with or without adjuvant at all timepoints (p > 0.05) except at day 35 (p < 0.001)(Fig. 3B). Despite high antibody titres towards the immunogen by the end of immunisations, a large proportion of this response was evidently directed against the VLP itself, with no significant difference determined between specific VLP-peptide or VLP only antibodies against each of the immunogens (Fig. 4, Fig. 1:12500 data point, p > 0.05 for all). In order to delineate how much of the response was directed towards peptide (including SpyTag) and not just mi3-SC, the ELISA experiments were repeated with peptide only instead of whole immunogen. Given the limited variation in rabbit pairs across the experimental groups, IgG from experimental pairs were pooled for subsequent analysis.

**Fig. 5:**
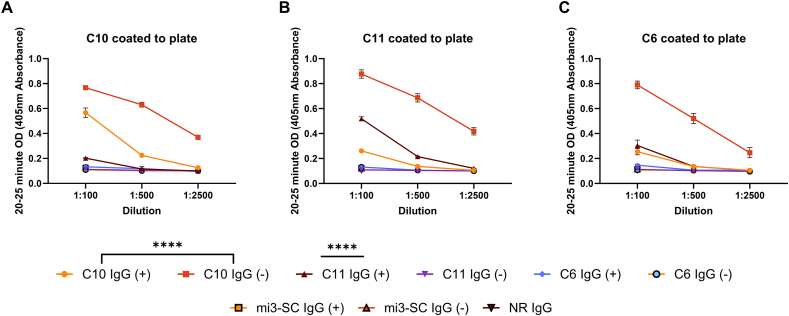
**Peptide specific recognition of rabbit IgG by ELISA.** The IgG of experimental pairs were tested against each of the consensus peptides at a series of dilutions to determined recognition of immunising peptide as well as potential cross-recognition (n = 2, each immunogen had a pair with or without adjuvant). The (+) denotes rabbits who received immunogen plus adjuvant, the (−) refers to the rabbits who received immunogen alone. A naïve rabbit IgG pool (n = 8) was tested against each peptide at a 1:100 dilution, and IgG from rabbits immunised with mi3-SC alone (n = 2, with or without adjuvant) was also tested against each peptide at a 1:100 dilution. Each sample was tested in duplicate, and data points show mean ± standard deviation of results for the experimental pair. Statistical results demonstrate analysis between the antibody verses each peptide against the corresponding controls at 1:100 dilution.

Antibodies raised against the C10 peptide demonstrated a good response against C10 (Fig. 5A p < 0.0001 verses naïve control for groups with and without adjuvant), and also had high cross reactivity to the C6 and C11 peptides (Fig. 5B–C, p < 0.0001 verses naïve control for groups with and without adjuvant). Antibodies raised against the C11 peptide with adjuvant also had a good response to their own peptide (Fig. 5B, p < 0.0001 compared to naïve control), and cross reactivity to C10 and C6 (p < 0.0001 compared to respective naïve controls) albeit at lower titres than observed with the C10 generated antibodies. However, C11 antibodies raised without adjuvant did not recognise peptides above their respective naïve controls (p > 0.05 for all three peptides). Antibodies raised against C6 had poor recognition across the board (Fig. 5A–C). Thus, further analysis of C6 antibodies was not considered, although this confirmed that antibody recognition of C10 and C11 was peptide specific and not directed against the SpyTag sequence. The peptides were not recognised by antibodies raised against mi3-SC only, which were statistically the same as the naïve controls (p > 0.05).

**Fig. 6:**
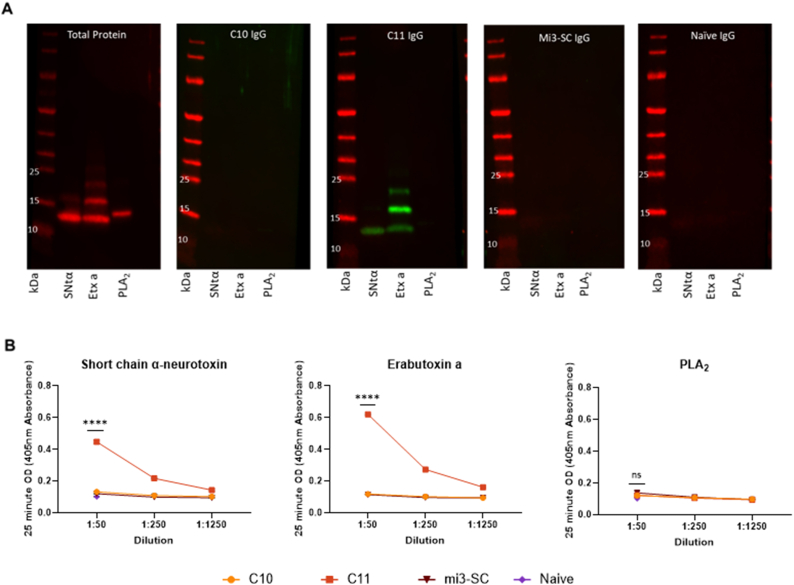
**Immunoblot and ELISA recognition of whole toxin by rabbit IgG.** The recognition of Short neurotoxin 1 from *N. pallida* (SNta), Erabutoxin a from *L. semifasciata* (Etx a) and a phospholipase A2 from *N. nigricollis* (PLA_2_) by IgG from rabbits immunised with C10-mi3-SC and C11-mi3-SC was tested by immunoblot (A) and ELISA (B). IgG was pooled from rabbits who received the same immunogen but may have received this with or without adjuvant (n = 4, n = 8 for naïve control). A) Immunoblots were performed in parallel with approximately 2 μg of each toxin loaded per lane. A total protein blot is shown as a loading control, as well as a blot probed with pooled naïve rabbit and mi3-SC only IgG. All IgG blots were tested with 1:1000 IgG dilution. B) IgG:toxin recognition was also tested in an ELISA format against 100 ng of each toxin with IgG dilutions of 1:50, 1:250 and 1:1250 for C10-mi3-SC and C11-mi3-SC IgG, and 1:50 for naïve and mi3-SC only IgG. Each sample was tested in duplicate, and data points show mean ± standard deviation. Statistical results demonstrate analysis between the antibody verses the toxin against the corresponding naïve controls at 1:100 dilution.

Next, the ability of C10 and C11 antibodies to recognise whole toxins was evaluated by immunoblot with a small number of purified whole toxins. sc3FTX from *N. pallida* and a sc3FTX (erabutoxin a) from *L. semifasciata* demonstrated that C11 antibodies, but not C10 antibodies, could recognise sc3FTx by western blot, and that this was toxin family specific given no recognition of the PLA_2_ (Fig. 6A). These results were confirmed by ELISA (Fig. 6B), with no recognition of PLA_2_ (no significant difference to naïve controls, p > 0.05) but strong recognition of the two sc3FTXs by C11 (p < 0.0001 verses naïve controls) providing support that C11 IgG may be able to bind to toxins in their conformational structure. As C10 antibodies were unable to recognise the toxins above naïve control for both toxins (p > 0.05 verses erabutoxin a naïve control, p = 0.0116 against sc3FTX *N. pallida* naïve control), further analysis of these samples was discontinued.

**Fig. 7:**
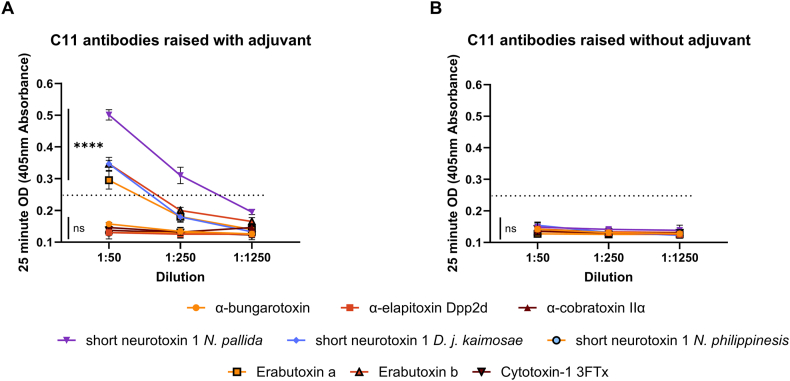
**The recognition of whole toxins by anti-**C**11 antibodies.** The ability of anti-C11 antibodies raised with (A) or without (B) adjuvant to recognise whole toxins was tested in an expanded toxin panel with IgG dilutions of 1:50, 1:250 and 1:1250. The panel consisted of 5 short chain neurotoxins (Short neurotoxin 1 from *N. pallida, D. j. kaimosae* and *N. philippinesis,* and Erabutoxin A and B from *L. semifasciata*), 3 long chain neurotoxins (α-bungarotoxin from *Bungarus multicintus,* α-elapitoxin Dpp2d from D. polylepis, and Alpha-cobratoxin, IIα from *N. kaouthia*) and a cytotoxic 3FTx (Cytotoxin-1 3FTx from *N. pallida*). The dotted line indicates the maximum OD of naïve and mi3-SC only IgG (1:50 dilution) against the different toxins (individual results presented in Supplementary File S2, Fig. S1). Each sample was tested in duplicate, and data points show mean ± standard deviation of results for the experimental pair. Statistical results demonstrate analysis between the antibody verses the toxin against the corresponding naïve controls at 1:100 dilution.

To further evaluate whole toxin recognition by C11 antibodies, and how specific this was to short chain toxins, a panel of 9 toxins was designed to include both sc3FTx and alpha long chain 3FTxs (lc3FTx) as well as a cytotoxic 3FTX (c3FTx). The response to whole toxins was short chain specific, with native short neurotoxins from *N. pallida* and *D. j. kaimosae* as well as erabutoxin a and b from *L. semifaciata* being recognised by antibodies raised with C11-mi3-SC plus adjuvant (Fig. 7A, p < 0.0001 for all toxins verses corresponding naïve and mi3-SC only controls), but no long chain or cytotoxic 3FTx recognition (p > 0.05). However, the short neurotoxin from *N. philippinesis* was not recognised by these same antibodies, demonstrating no significant difference to naïve, mi3-SC only, or non-sc3FTx toxins (p > 0.05). There was no recognition of any whole toxin by antibodies raised by the same immunogen without the presence of adjuvant (Fig. 7B–p > 0.05 verses corresponding naïve for all toxins). Individual results for naïve and mi3-SC only IgG against each toxin can be seen in Supplementary File S2, Fig. S1, although the maximum OD value of these controls is presented as a dotted line on Fig. 7.

### 3.3 Murine pre-incubation model

**Fig. 8:**
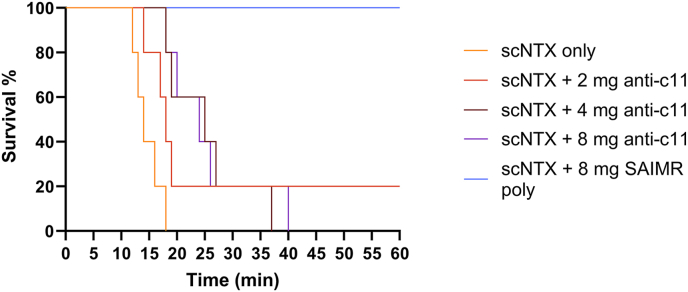
**Kaplein-Meyer survival curves to show the capability of anti-**C**11 antibodies to neutralise the lethal effects of a short chain alpha 3FTX purified from the venom *N. pallida* in a murine pre-incubation model.** All groups received a toxin challenge consisting of 4 μg (approximately 2 x LD_50_) of short neurotoxin 1 purified from the venom *N. pallida* in PBS which had been pre-mixed and incubated for 30 min at 37 °C with the following: PBS only (black, no intervention control), 2 mg of anti-C11 antibodies (pink), 4 mg of anti-C11 antibodies (blue), 8 mg of anti-C11 antibodies (dark purple), or 8 mg of SAIMR polyvalent (light purple) (n = 5 per group). (For interpretation of the references to colour in this figure legend, the reader is referred to the Web version of this article.)

The capability of anti-C11 antibodies to neutralise the lethal effects of a sc3FTx purified from the venom *N. pallida* was evaluated in a murine pre-incubation model (Fig. 8). Mice challenged with 4 μg of toxin-only demonstrated typical neurotoxic envenoming characteristics (progressive reduction in movement, laboured breathing and eventual loss of self-righting reflex) and reached humane endpoints with a median time of 14 min (range 12–18 min). Mice receiving the same toxin challenge but pre-incubated with anti-C11 antibodies demonstrated increases in time to humane end point, which manifested as delayed onset of the typical overt pathological symptoms of neurotoxic envenoming described above. Mice receiving 2 mg of anti-C11 antibodies displayed slightly increased, but not statistically significant (p = 0.07) median time to humane endpoint to 18 min (range 14–60 min) as compared to the toxin only group, with a single mouse surviving until the end of experiment. Increasing the dose of anti-C11 antibodies to 4 mg significantly increased median time to humane end point to 25 min (range 18–37 min) (p = 0.004 as compared to toxin only challenge), however, further increasing the dose to 8 mg of anti-C11 antibodies, whilst still significantly different compared to toxin only challenge (p = 0.004) did not lead to increased median time to humane endpoint (24 min, range 18–40 min) as compared to the group receiving 4 mg of anti-C11 antibodies. Mice challenged with toxin preincubated with 8 mg of SAIMR polyvalent antivenom did not demonstrate any overt signs of envenoming and all survived to the end of the experiment (60 min).

## 4 Discussion

Antivenoms have inherent deficiencies which are a direct result of the use of crude venom as the starting immunogen in their manufacture. Not all components of crude venom are medically relevant, resulting in the production of medically redundant anti-venom antibodies (Tan et al., 2016), and components which are medically relevant are often classed as being poorly immunogenic (León et al., 2011; Pruksaphon et al., 2022; Chan et al., 2023), resulting in weakly potent responses. The rational design of antigens for use in antivenom manufacture is a promising avenue of research which may circumvent these issues, by focusing and boosting antibody development in antivenom manufacturing animals to only medically relevant venom components (Bermúdez-Méndez et al., 2018; Ratanabanangkoon et al., 2020; Harrison, 2004).

One particular approach is the use of VLPs decorated with linear epitopes of medically important toxins, which we previously demonstrated could generate broadly reactive anti-venom antibodies without the use of crude venoms (Menzies et al., 2022). In our previous study, we utilised HBcAg VLPs genetically fused to various linear epitopes. A major finding and ultimately a drawback of that study was the difficulty in expression of each individual VLP-epitope fusion, with many being unstable or providing poor yields on expression, severely limiting the translational capability of the approach. In this study we modified the approach, through the use of mi3-SC VLPs which enable a “plug and play” format (Bruun et al., 2018), allowing any antigen with an appropriate tag to be irreversibly coupled to a single universal VLP, thus negating extensive expression and optimisation trials of individual VLP-epitope fusions. The linear epitopes examined in this particular study were chemically synthesised, ordered online and delivered in approximately three weeks ready for coupling and investigation.

Previous approaches in design of linear epitopes for toxin development have used either *in silico* prediction tools and/or low resolution/low throughput peptide arrays (Ramos et al., 2016; Krause et al., 2020). By using high density peptide arrays representing all known sc3FTx sequences at the time, we were able to identify six linear epitope regions frequently recognised by geographically and species distinct antivenoms. Due to the diversity of antivenoms and species from which the sc3FTx sequences were acquired, we inferred that these specific epitopes were capable of generating broadly recognising anti-sc3FTx antibodies and therefore rational targets for development of broadly neutralising anti-sc3FTx sera. Examining the responses of rabbits immunised with VLPs coupled to the three most frequently recognised epitopes identified in the peptide arrays demonstrated that two of the three epitopes failed to produce antibodies capable of recognising toxins, while antibodies raised against epitope C11 were successful in recognition of conformational toxins from multiple snake genera. As it is clear that antivenoms contain antibodies which readily recognise C10 and C6 epitopes, it is difficult to speculate with any confidence as to why the C10 and C6 immunogens failed to elicit antibodies in rabbits which were capable of recognising toxins. The generation of consensus sequences could have affected key residues necessary for binding, or in the instance of C6, there could have been species specific differences in the antibody response of rabbits compared to antivenom manufacturing animals such as horses and sheep. In contrast, there any many instances were immunorecognition *in vitro* does not correlate to neutralisation *in vivo,* quite often because the antibodies target epitopes unrelated to functional activity. This is most commonly observed in the screening of monoclonal antibodies, whereby a whole pipeline of candidates results in a much small number of viable therapeutic options capable of neutralisation (Laustsen et al., 2018; Rimbault et al., 2023; Sørensen et al., 2023).

Although able to recognise a range of sc3FTx from multiple genera and locations, anti-C11 antibodies were unable to recognise sc3FTx-1 from *N. philippinensis*. The failure to recognise this sc3FTX is possibly due to a divergent alanine residue (corresponding to the 11th amino acid residue) within the *N. philippinensis* sc3FTx sequence. This variant residue was previously thought to be unique to *N. philippinensis* (Hauert et al., 1974), although it has recently been demonstrated to be present in further species but is ultimately rare across *Naja* sc3FTxs (Tan et al., 2019). The presence of single AA substitutions that can drastically alter the recognition of toxins by antivenoms, which has previously been studied in detail, suggests that use of multiple variants of epitopes are likely to be required to generate broad recognition and neutralisation through such approaches. With this in mind, the flexibility of the “plug and play” VLP system could further benefit development of antivenoms. For instance, it is possible to use multiple antigens on the same VLP, generating so-called mosaic VLPs which have been generated for eliciting anti-viral antibodies (Cohen et al., 2021), or could ‘focus’ existing immune responses generated with crude venom towards specific toxins (Alomran et al., 2022). Recently, with the introduction and widespread availability of commercial bacterial strains engineered to be capable of expressing heavily disulphide-bonded proteins, the expression of recombinant and functional low molecular weight toxins, particularly so-called consensus toxins, have also started to become routine (de la Rosa et al., 2019; Guerrero-Garzón et al., 2018; Rivera-de-Torre et al., 2024). Due to the small size of the SpyTag required for coupling toxins to the SpyCatcher on mi3-SC VLPs, it is not unfeasible that the VLP approach outlined here could be adapted to such recombinant toxins, enabling the generation of widely regarded superior conformational epitopes.

Virus-like particles, by their inherent viral like shape, are highly immunostimulatory, potently activating both innate and adaptive immune responses, and are therefore proposed to be self-adjuvating molecules (Lua et al., 2014; Noad and Roy, 2003; Sharma et al., 2020). Due to welfare concerns generated by FCA, which frequently results in local complications in immunised animals, alongside the high proportion of redundant antibodies in antivenoms (Casewell et al., 2010), limiting the exposure of non-snake venom antigens, such as those in FCA, during the immunisation process is desirable. It is therefore disappointing that only animals which received C11-mi3-SC in adjuvant elicited toxin recognising antibodies, whilst C11-mi3-SC without adjuvant failed to do so.The majority of studies utilising mi3-SC VLP have been performed in mice (Pascha et al., 2024; Tan et al., 2021; Kang et al., 2021), however its use in rabbits and their host response to mi3-SC remains uncharacterised. Another study vaccinating rabbits with a different VLP-based vaccine for Epstein–Barr virus (EBV) found it unable to prevent subsequent EBV infection, and suggested adjuvants remain warranted (Reguraman et al., 2023). However, even within studies utilising mice, there has often been inconsistencies between individual immune responses and the necessity for adjuvant or the merits of different VLP platforms (Zegeye et al., 2022). Furthermore, the majority of commercial VLP-based vaccines continue to incorporate adjuvants in order to maximise the immunity elicited by the vaccine (Cimica and Galarza, 2017). Together, this suggests that strong adjuvants are likely to continue to be required if using mi3-SC VLPs for antivenom development.

We have demonstrated that immunisation with a single linear epitope displayed on a VLP can partially neutralise toxin pathology. However, whilst the antibodies did result in a significant increase in time to the onset of neurotoxic symptoms, they ultimately did not prevent lethality. Despite this, the partial neutralising response of anti-C11 peptides is notable and needs to be viewed in context with broader realities of toxin neutralisation. Similar experiments with anti-toxin monoclonal antibodies use doses in the range of 27–85 mg/kg to achieve full neutralisation (Khalek et al., 2024). The doses of polyclonal antibody here range from 50 to 400 mg/kg, assuming 90% of polyclonal serum is not toxin-specific (Casewell et al., 2010), we can estimate the proportion of the dose of anti-C11 antibodies to be 5–40 mg/kg. Furthermore, the majority of polyclonal antivenoms are generated through a hyper-immunisation schedule over an extended period, allowing subsequent affinity maturation and an increase in neutralising potency over time (de la Rosa et al., 2019; León et al., 2021). The anti-C11 serum here was generated in 2.5 months with six immunisations following a standard rabbit polyclonal antibody production schedule, in contrast to many large animal models that often extend to 3–6 months or beyond (de la Rosa et al., 2019; León et al., 2021; Alomran et al., 2021; Chotwiwatthanakun et al., 2001; El-Kady et al., 2009; Smith et al., 1992). Likewise, mAbs such as those referenced above, are generated after repeated rounds of panning and selection in order to choose highly potent broadly neutralising antibodies. The immunisation doses of 50 μg were also 20x lower than other leporine models used to generate snake antisera with crude venom (Gómez et al., 2023, 2024), which still resulted in poor neutralising capabilities *in vivo* in some instances*,* particularly for *Dendroaspsis* species which are dominated by 3FTxs. In light of this, we suspect that with increased doses and extended immunisation schedules, to allow for affinity maturation and increased titres, this approach would be able to generate a polyclonal sera with substantially increased potency.

In summary, this study provides proof of principle that VLP plug and play technology is capable of flexibly developing experimental toxin-specific serotherapy and provides exciting rational for further exploration of this approach to rationally design and generate immunogens for antivenom development.

## Ethical statement

Polyclonal antibody production was performed by Antibody Production Services (a division of Life Science Group Ltd., UK) in accordance with the UK Animals (Scientific Procedures) Act 1986. Murine animal experiments were conducted under protocols approved by the Animal Welfare and Ethical Review Boards of the Liverpool School of Tropical Medicine and the University of Liverpool, as per project license PP2669304 approved by the UK Home Office in accordance with the UK Animal (Scientific Procedures) Act 1986.

## CRediT authorship contribution statement

**Rebecca J. Edge:** Writing – original draft, Visualization, Project administration, Methodology, Investigation, Formal analysis. **Amy E. Marriott:** Writing – review & editing, Investigation. **Emma L. Stars:** Writing – review & editing, Investigation. **Rohit N. Patel:** Writing – review & editing, Resources, Investigation. **Mark C. Wilkinson:** Writing – review & editing, Resources, Investigation. **Lloyd D.W. King:** Writing – review & editing, Resources. **Julien Slagboom:** Writing – review & editing, Resources. **Choo Hock Tan:** Writing – review & editing, Resources. **Kavi Ratanabanangkoon:** Writing – review & editing, Resources. **Simon J. Draper:** Writing – review & editing, Resources. **Stuart Ainsworth:** Writing – original draft, Visualization, Supervision, Methodology, Funding acquisition, Formal analysis, Data curation, Conceptualization.

## Declaration of competing interest

The authors declare the following financial interests/personal relationships which may be considered as potential competing interests: Simon J Draper reports a relationship with SpyBiotech that includes: board membership and equity or stocks. If there are other authors, they declare that they have no known competing financial interests or personal relationships that could have appeared to influence the work reported in this paper.

## Data Availability

Data will be made available on request.
