# Supplementary material for: Plug and play virus-like particles for the generation of anti-toxin antibodies
Source: Toxicon X. 2024 Aug 26;23:100204. doi: 10.1016/j.toxcx.2024.100204 (PMC11401359; doi:10.1016/j.toxcx.2024.100204)
Supplement: Multimedia component 2 [file mmc2.docx]

**Table S1. Equine antibodies and antivenoms used to probe peptide microarrays.** Further details on the format, concentration and snake venom indicated to be neutralises by each based off product inserts, manufacturer websites or literature.

| **Antivenom** | **Format** | **Concentration** | **Indication (source)** |
| --- | --- | --- | --- |
| Naïve Horse IgG | equine IgG | 1 mg/ml | NA |
| SAIMRpoly | equine F(ab)'2 antivenom | 79.5 mg/ml | *Bitis arietans, B. gabonica, Dendroaspsis angusticeps, D. jamesoni, D. polylepis, Hemachatus hemachatus, Naja annulifera, N. melanoleuca, N. mossambica, N. nivea* (1) |
| FAVafrique | equine F(ab)'2 antivenom | 77.5 mg/ml | *Bitis arietans, B. gabonica, D. jamesoni, D. polylepis, D. viridis, Echis leucogaster, E. ocellatus, Naja haje, N. melanoleuca, N. nigricollis* (1) |
| PremiumAfrica | equine F(ab)'2 antivenom | 34 mg/ml | *Bitis arietans, B. gabonica, B. nasicornis, B. rhinoceros, Dendroaspsis angusticeps, D. jamesoni, D. polylepis, D. viridis, Echis cariatus, E. leucogaster, E. ocellatus, Naja nigricollis, N. haje, N. melanoleuca* (1) |
| PremiumIndia | equine F(ab)'2 antivenom | 30.7 mg/ml | *Naja naja, Bungarus caeruleus, Vipera russelii, Echis carinatus* (2) |
| TRC Neuro Poly | equine F(ab)'2 antivenom | 36.8 mg/ml | *Ophiophagus hannah, Naja kaouthia, Bungarus fasciatus, B. candidus* (Product packaging) |
| ICP anticoral | equine IgG antivenom | 28.5 mg/ml | *Micrurus* species |
| CSL poly | equine F(ab)'2 antivenom | 80.3 mg/ml | *Pseudonaja* spp. *Notechis scutatus, N. ater, Tropidechis carinatus, Austrelaps ramsayi, A. labialis, Hoplochephalus bungaroides, H. bitorquatus, H. stephensi, Pseudechis* spp. *Acanthophis* spp. *Oxyuranus* spp. (3) |
| EXPneuro | equine IgG | 1 mg/ml | 36 neurotoxic snakes across 10 genera and 4 continents (4) |

**Supplementary File S2. Native toxin purifications.** The methodology for native toxins purified from whole venom at Liverpool School of Tropical Medicine.

*Purification of neurotoxic three finger toxins*

For the isolation of the neurotoxins used in this study, venom was extracted from the following snake species maintained at the herpetarium facility at the Liverpool School of Tropical Medicine (UK), these are listed with species name and country of origin if not captive bred: *Naja pallida* (Tanzania), *Naja kaouthia* (captive bred), *Naja philippinensis* (captive bred), *Dendroaspis polylepis* (Tanzania) and *Dendroaspis jamesoni kaimosae* (Uganda). The protein components of the venoms were initially separated using cation exchange chromatography. For this, freeze-dried venom (10 mg) was resuspended in 2 mL of 50 mM sodium phosphate, pH 6.0 and applied to a 4.7 mL HiScreen Capto S column (Cytiva) equilibrated in the same buffer. Elution was carried using a 15-column volume gradient of 0 - 0.6 M NaCl in 50 mM sodium phosphate, pH 6.0. The flow rate was 0.6 mL/min and elution was monitored at 280 nm. The unbound material was retained and 1 mL fractions were collected from the start of the NaCl gradient. SDS-PAGE was used to determine purity of the fractions from the main peaks and muscle-type nicotinic acetylcholine receptor (nAChR) activation assays were carried out on all 3FTx-containing peaks as described in Patel et al., 2023 (5)**.** Those that were determined to be active via inhibition of nAChR activation were further purified using RP-HPLC. For this 20-50 µg protein was loaded onto a BioBasic C4 column [100 mm x 0.2 mm, Thermo Fisher] and eluted using the following gradient of acetonitrile in 0.1% (v/v) trifluoroacetic acid: 0-32% in 40 mins; 32-70% in 5 mins. Elution was monitored at 214 nm. Eluted proteins were again tested for inhibition of nAChR activation and those which were chosen for testing against VLP-epitope derived antibodies were prepared for identification by mass spectrometry. Thus, 10 µg of the protein was dried, reduced and carboxymethylated and then 0.1 µg of sequencing grade trypsin was added and left overnight.

Tryptic digests of the purified toxins were analysed with microLC separations using an UltiMate 3000 RPLCnano system (ThermoFischer Scientific, Ermelo, The Netherlands). 0.1 μL of sample was injected and separated on an Kinetex 2.6u XB-C18 LC Column (150 mm × 0.3 mm) with a particle size of 2.6 μm and a pore size of 100 Å at a flow rate of 6 μL/min. The mobile phases comprised of eluent A (98% water, 2% ACN, 0.1% FA) and eluent B (98% ACN, 2% water, 0.1% FA). The used gradient for the separation of the digests was: 3 min isocratic separation at 1% B, linear increase to 40% in 7.5 min followed by a linear increase to 90% in 0.1 min, isocratic separation at 90% B for 0.7 min, linear decrease to 1% B in 0.2 min and finally the column was equilibrated for 3.7 min at 1% B. Mass detection was conducted using a Sciex 7600 ZenoTOF instrument (Framingham, MA, USA). The instrument utilised the OptiFlow 1–50 μL Micro/MicroCal ion source in positive mode. The method parameters for the ZenoTOF were as follows: the chosen workflow targeted peptides with a duration of 13 minutes. Ionization commenced at 2 minutes and ceased at 13 minutes. The following source and gas parameters were used: curtain gas was set to 35, CAD gas was set to 7, ion source gas 1 was set to 15 psi and ion source gas 2 to 20 psi, temperature was set to 150 °C and the column temperature to 30 °C. IDA experiment settings were as follows: spray voltage 4500 V, TOF mass range: 400–1500 Da, accumulation time: 0.1 s, declustering potential: 80 V, DP spread: 0 V, collision energy:10 V, CE spread: 0 V, time. TOF MS Advanced experiment settings used were: bins to sum: 8, channels 1–4: selected. The IDA criteria were: peptide workflow, maximum candidates ions: 45, intensity threshold exceeds of 300 counts/s, no dynamic background subtract, exclusion of former candidate ions for 6 s and after one occurrence. Dynamic CE for MS/MS was used. Maximum CE was set to 80 V and the minimum to 5 V. The selected charge states were 2 to 5 and the selected isotope was monoisotopic. In advanced IDA criteria a mass tolerance of ±50 mDa was set. TOF MS/MS settings were as follows: fragmentation mode: CID, TOF mass range: 100–2000 Da, accumulation time: 0.01 s, declustering potential: 80 V, spread: 0 V, collision energy spread: 0 V, Q1 resolution set to unit, zeno pulsing on. TOF MS/MS Advanced experiment settings were: zeno threshold:100000 cps, time bins to sum: 8, channels 1–4: on.

The obtained mass spectrometry data was processed into mgf files by using ProteoWizard's msConvert. The following search parameters were used: Instrument type: ESI-QUAD-TOF, Digestion enzyme: Trypsin, Allowing one missed cleavage, Carbamidomethyl on cysteine as a fixed modification, deamidation and oxidation on methionine as variable modifications, Fragment mass tolerance: ±0.05 Da, and ±0.2 Da peptide for mass tolerance. Database searches were done by using the Uniprot database containing only Serpentes accessions.

*Purification of cytotoxic three finger toxins and Phospholipase A2*

Cytotoxin-1 3FTx (*Naja pallida,* Tanzania*)* and PLA_2_ (*Naja nigricollis*, Tanzania) were purified and identified by mass spectrometry analysis as described by Bartlett *et al*., 2023 (6). The acidic and basic forms of PLA _2_ were previously pooled at a 1:1 ratio and the pool was used in the present study to test the class specificity of VLP-epitope derived antibodies.

**Table S2. Consensus linear epitope sequences.** The sequence, as well as the full synthesis sequence including glycine serine linker (GSGGSGGSG) and c-terminal SpyTag (GAHIVMVDAYKPTK), of the epitopes used for immunisation in rabbits.

| **Name** | **Epitope (length)** | **Sequence (final length)** |
| --- | --- | --- |
| c10 | NVKPGIKLNCCTTDRCN (17) | NVKPGIKLNCCTTDRCNGSGGSGGSGAHIVMVDAYKPTK (39) |
| c11 | ETNCYKKTWSDHRGT (15) | ETNCYKKTWSDHRGTGSGGSGGSGAHIVMVDAYKPTK (37) |
| c6 | NQQSSQPKTTKSCS (14) | NQQSSQPKTTKSCSGSGGSGGSGAHIVMVDAYKPTK (36) |

**
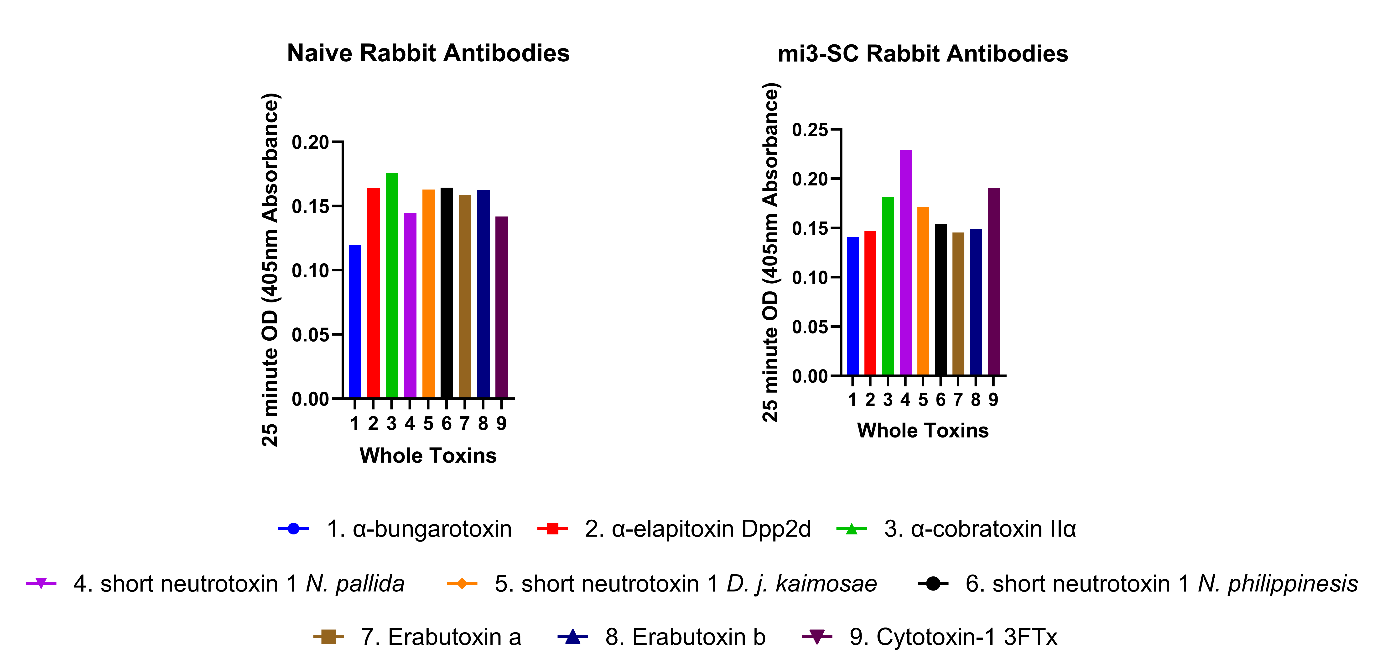
**

**Figure S1.** **Naive and VLP only controls for the recognition of whole toxins by anti-c11 antibodies.** A naïve rabbit IgG pool (n=8) and pooled IgG from rabbits immunised with mi3-SC alone (n=4) were tested against each toxin at a 1:50 dilution. Each sample was tested in singlet, and bars show optical density measured at 405nm at 25 minutes into development. The dotted line indicated on Figure 7 demonstrates the maximum OD of these naïve and mi3-SC only IgG results (mi3-SC antibodies against short neurotoxin 1 from *Naja pallida*).

**References**

1. Potet J, Smith J, McIver L. Reviewing evidence of the clinical effectiveness of commercially available antivenoms in sub-Saharan Africa identifies the need for a multi-centre, multi-antivenom clinical trial. PLOS Neglected Tropical Diseases. 2019 Jun 24;13(6):e0007551.

2. PS&V. Premium Serums. [cited 2024 Apr 3]. Snake Venom Antiserum I.P. Available from: https://www.premiumserums.com/product2.html

3. CSL. CSL Antivenom Handbook Antivenoms. 2013 [cited 2024 Apr 3]. CSL Antivenom Handbook - Polyvalent Snake Antivenom. Available from: http://www.toxinology.com/generic_static_files/cslavh_antivenom_polyvalen.html

4. Ratanabanangkoon K, Tan KY, Pruksaphon K, Klinpayom C, Gutiérrez JM, Quraishi NH, et al. A pan-specific antiserum produced by a novel immunization strategy shows a high spectrum of neutralization against neurotoxic snake venoms. Sci Rep. 2020 Jul 9;10(1):11261.

5. Patel RN, Clare RH, Ledsgaard L, Nys M, Kool J, Laustsen AH, et al. An in vitro assay to investigate venom neurotoxin activity on muscle-type nicotinic acetylcholine receptor activation and for the discovery of toxin-inhibitory molecules. Biochem Pharmacol. 2023 Oct;216:115758.

6. Bartlett KE, Hall SR, Rasmussen SA, Crittenden E, Dawson CA, Albulescu LO, et al. Dermonecrosis caused by spitting cobra snakebite results from toxin potentiation and is prevented by the repurposed drug varespladib [Internet]. bioRxiv; 2023 [cited 2024 Mar 28]. p. 2023.07.20.549878. Available from: https://www.biorxiv.org/content/10.1101/2023.07.20.549878v2
